# Genetic diversity analysis in *Plectranthus edulis* (Vatke) Agnew populations collected from diverse geographic regions in Ethiopia using inter-simple sequence repeats (ISSRs) DNA marker system

**DOI:** 10.1186/s40709-019-0100-3

**Published:** 2019-09-09

**Authors:** Medhin Gebrehiwet, Teklehaimanot Haileselassie, Fekadu Gadissa, Kassahun Tesfaye

**Affiliations:** 1 Department of Biotechnology, Aksum University, P.O. Box 1010, Aksum, Tigray Ethiopia; 2 Institute of Biotechnology, Addis Ababa University, P. O. Box 1176, Addis Ababa, Ethiopia; 3 Biology Department, Madda Walabu University, P. O. Box 247, Bale Robe, Ethiopia; 4 Ethiopian Institute of Biotechnology, Ministry of Science and Technology, P.O. Box 32853, Addis Ababa, Ethiopia

**Keywords:** Ethiopian potato, Genetic diversity, ISSR, Molecular marker, *Plectranthus edulis*

## Abstract

**Background:**

*Plectranthus edulis* (Vatke) Agnew (Lamiaceae), locally known as Ethiopian potato *syno.* Ethiopian dinich, is one of the native Ethiopian edible tuber crops that has been significantly contributing to household food security for millions of subsistence farmers. However, its current production is declining to the extent of total extinction from several administrative regions where it used to be widely cultivated. It is one of the less researched crops regardless of being indigenous and its contribution to food security during time of scarcity. Therefore, we intended to assess the level of genetic diversity in 67 accessions, representing nine populations that were collected from diverse agro-ecologies in the country, using ISSR markers and hence, to generate a baseline information that assists marker assisted breeding, conservation and germplasm management efforts.

**Results:**

In the present study, ten polymorphic ISSR markers were screened and optimized, that generated an average of 7.4 scorable bands per marker and revealed high overall percent polymorphism (95%), Nei’s gene diversity (h = 0.40) and Shannon index (I = 0.62) suggesting ISSR’s effectiveness in detecting high levels of genetic diversity. A considerably high overall populations gene diversity (Nei’s) (h = 0.32) and Shannon index (I = 0.47) were observed, revealing high potential of the populations for further breeding and conservation efforts particularly for population from Gurage administrative zone, which showed the highest values. Similarly, estimation of pairwise genetic distance revealed the importance of cross breeding population from Awi administrative zone to the rest populations. Analysis of hierarchical molecular variance (AMOVA) showed higher levels of genetic differentiation within populations (92%), and collection regions (94%) suggesting that either clonal mode of propagation in the crop or farmers selection pressure for important agronomic traits or both maintained the original heterozygosity in the crop. UPGMA phylogenetic analysis did not strictly group the populations based on their geographic region of origin, which could be attributed to the widely practiced tuber exchange and hence continuous human mediated exchange of genetic material and sharing of the same genetic base among the geographic regions.

**Conclusions:**

The ISSR markers used in the present study were effective in revealing extent and patterns of genetic diversity in *P. edulis* populations. However, it is important to couple them with agro-morphological traits or codominant molecular markers to get more reliable information for use in breeding and conservation. Several of the potential administrative zones we covered are useful for *P. edulis* diversification and conservation. However, the crop is currently highly marginalized and this led to rapid decline in population size and loss of valuable agronomic traits. To address this challenge, there is an urgent need to take counteractive measures.

## Background

*Plectranthus edulis* (Vatke) Agnew is locally known by several vernacular names among which, Ethiopian potato *syno.* Ethiopian dinich, is frequently used by the scientific community. It is an ancient tuber crop and native to Ethiopia. The crop belongs to family Lamiaceae, subfamily Nepetoideae, tribe Ocimeae, and genus *Plectranthus* [1, 2]. It has a wide range of adaptations and used to be widely cultivated in the Central, Southern, Western, Northwestern and South-Western parts of Ethiopia. It is also reported to be found in warmer African countries such as Kenya, the Democratic Republic of Kongo, and Uganda, mainly in a wild form [3, 4]. The crop is one of the four economically important tuber crops of the genus *Plectranthus*, such as *P. esculentus* (Livingstone potato)*, P. parviflorus* (Sudan potato) and *P. rotundifolius* (Madagascar potato) [5–7].

In Ethiopia, *P. edulis* is primarily cultivated for food and as a source of income for millions of subsistence farmers, particularly in the country’s densely populated highlands and semi-highlands. The crop is also widely used as a folk medicine and a source of nectar for honeybee [8, 9]. However, production of the crop is currently declining to the extent of total extinction from several areas where it used to be widely cultivated. The decline in cultivation could be attributed to the current restricted distribution of the crop, low attention from the local scientific community, lack of awareness among younger farmers about the conservation and cultivation of the crop, research focus of the country that mainly targets cereals and commercial crops, poor shelf life of the crop, poor market opportunities, and introduction of exotic crops such as Irish potato to the area where it is cultivated [10]. Furthermore, the cultivation of the crop is currently restricted to elderly farmers mainly using marginal and degraded plots of land that are thought to be less important for other crops [11].

Genetic diversity, the total genetic variation in a species, could be assessed using a particular method or a combination of methods such as agro-morphological traits, biochemical and/or DNA marker(s) methods. Because of their ability to generate more reliable information, DNA molecular markers have been used for more than 30 years in estimating genetic diversity values [12–14]. ISSR is one of the DNA based marker systems that involves the amplification of DNA segment oriented in opposite direction between two identical microsatellites repeat regions. It is widely applicable in genetic diversity study of crop plants and is therefore an important tool for characterizing and conserving germplasm, particularly in the breeding and management of endangered, rare and non-market-oriented endemic species [15, 16].

So far, very limited research activities have been conducted on *P. edulis* focusing only on agro-morphological diversity, ontogeny, micro-propagation, phytochemistry and nutritional analysis. As far as we know, very limited work is available using molecular DNA data such as EST-SSR [17] and no report is available on evaluation of its genetic diversity using ISSR markers. This study was therefore conducted in view of assessing the extent of genetic diversity within and among *P. edulis* populations collected from diverse agro-ecological regions in Ethiopia using ISSR markers. The generated information could be used as baseline in the future to support marker assisted breeding, conservation and germplasm management of this crop.

## Methods

### Plant material

**Fig. 1:**
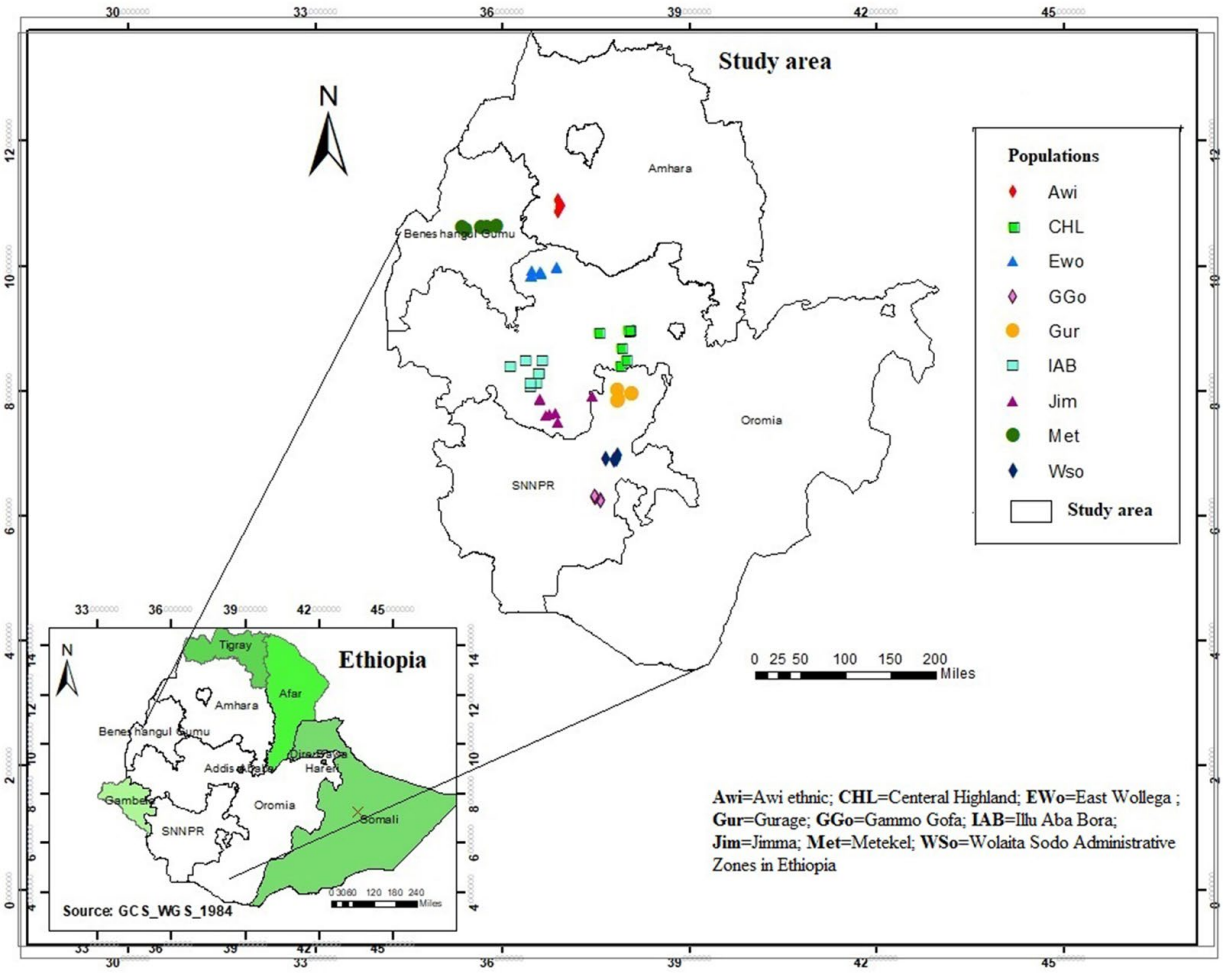
A map of Ethiopia with federal administrative regions (left down) showing *P. edulis* accessions collection administrative regions (left up). The map was constructed based on geographic coordinates and elevation data gathered from each collection sites using global positioning system (GPS)

Seeds of 67 *P. edulis* accessions, representing nine populations, were collected from the four main growing regions in the country (Table 1; Fig. 1). The seed samples for each accession were planted on separate pots filled with soil in a glasshouse at College of Natural Sciences, Addis Ababa University, Ethiopia. After planting, 5-week old young leave tissues were collected from five plants per accession and dried in a silica-gel filled zip-lockbag.

**Table 1:** List of *P. edulis* populations and the administrative regions, zones and woredas of collection used in the present study, along with their altitude, latitude and longitude

Population	Admin. Region	Admin. Zone	Admin. Woreda	Coll. Code	Altitude (m)	Latitude (dd)	Longitude (dd)
*Awi*	Amhara	Awi	Banja	PE001	2554	10.937	36.912
				PE002	2557	10.938	36.914
				PE003	2643	10.973	36.948
			Fagta Lekoma	PE004	2555	11.058	36.893
				PE005	2590	11.044	36.891
			Ankesha Goagsa	PE006	2380	10.873	36.896
				PE007	2369	10.874	36.893
*Met*	Benshangul Gumuz	Metekel	Wenbera	PE008	2505	10.635	35.892
				PE009	2427	10.623	35.346
				PE010	2517	10.583	35.400
				PE011	2483	10.614	35.646
				PE012	2501	10.613	35.745
*CHL*	Central Highland, Oromia	Southwest Shewa	Darian	PE013	2599	8.694	37.896
			Goro	PE014	1828	8.412	37.872
			Woliso	PE015	1972	8.506	37.965
		West Shewa	Cheliya	PE016	2924	9.109	37.399
				PE017	2333	8.958	37.538
				PE018	2469	8.973	38.007
			Dandi	PE019	2441	8.979	38.016
				PE020	2446	8.981	38.020
				PE021	2445	8.978	38.024
*IAB*	Oromia	Ilu Aba Bora	Metu	PE022	1694	8.284	36.587
				PE023	1714	8.285	36.585
				PE024	1696	8.285	36.587
			Alle	PE025	1794	8.134	36.553
			Didessa	PE026	1915	8.485	36.642
				PE027	1879	8.072	36.451
				PE028	2096	8.135	36.452
			Bedele	PE029	1947	8.393	36.124
				PE030	1901	8.483	36.370
*Jim*		Jimma	Goma	PE031	1605	7.868	36.593
				PE032	1621	7.874	36.599
			Seka Che	PE033	1806	7.605	36.690
			Dedo	PE034	2181	7.505	36.892
				PE035	2228	7.502	36.890
			Jimma	PE036	1835	7.636	36.763
			Sokoru	PE037	1719	7.655	36.845
				PE038	1987	7.911	37.435
				PE039	1927	7.919	37.431
*Ewo*		East Wollega	Gida Ayana	PE040	1991	9.887	36.613
				PE041	2126	9.920	36.594
				PE042	2087	9.895	36.628
			Limmu	PE043	2166	9.927	36.473
				PE044	2149	9.846	36.459
				PE045	2151	9.846	36.458
			Kiramu	PE046	2163	9.983	36.870
				PE047	2141	9.990	36.870
*GGo*	SNNPs	Gamo Gofa	Chencha	PE048	2678	6.261	37.582
				PE049	2705	6.262	37.579
				PE050	2696	6.246	37.564
			Dita	PE051	2625	6.302	37.496
				PE052	2602	6.301	37.488
				PE053	2623	6.299	37.485
				PE054	2513	6.309	37.487
*Gur*		Gurage	Endegagn	PE055	2261	7.844	37.837
				PE056	2445	7.848	37.848
				PE057	2382	7.857	37.856
			Enemorna Ener	PE058	2131	8.017	37.847
				PE059	2155	8.011	37.849
			Gumer	PE060	2878	7.964	38.063
				PE061	2929	7.961	38.067
				PE062	2906	7.958	38.071
*Wso*		Wolaita Sodo	Damot Gale	PE063	2012	6.961	37.842
				PE064	2159	6.917	37.818
			SodoZuriya	PE065	2215	6.880	37.792
				PE066	2188	6.901	37.821
			Damot Sore	PE067	2099	6.905	37.640

PE: *Plectranthus edulis*; Col.: collection; Admin.: administrative; SNNPs: South Nations Nationalities and Peoples; Seka che: Seka Chekorsa; dd: decimal degree geographic coordinate system

### Genomic DNA extraction, primer screening and optimization

Genomic DNA extraction and PCR amplification were performed at the Plant Genetics Research Laboratory, College of Natural Sciences, Addis Ababa University, Ethiopia. Following the CTAB (2% Cetyl Trimethyl Ammonium Bromide) protocol [18], approximately 0.5 g of silica gel dried and Restech mixer mill fine ground leaf powder was used for genomic DNA extraction with minor modifications such that the chloroform extraction step was repeated three times to yield high-quality DNA. The quality and quantity of DNA was checked using 1% w/v agarose gel and Thermo Scientific nanodrop spectrophotometer (NanoDrop 2000/2000c, Thermo Fisher Scientific, Wilmington, USA), respectively.

Twenty-five ISSR primers (Source: Primer kit 900 (UBC 900) obtained from University of British Columbia, Vancouver, Canada) were used during the initial screening for variability and reproducibility. Ten (seven di-nucleotides—five of which were anchored, two tri-nucleotides and one penta-nucleotide) polymorphic primers capable of generating reproducible bands were selected and optimized for the study (Table 2).

**Table 2:** List of ISSR primers along with their annealing temperature, respective sequences and amplification efficiency used during optimization

S/N	Primer	Annealing temperature (°C)	Primer sequences	Amplification efficiency
1	UBC-812	47 °C	(GA)8A	Excellent
2	UBC-817	48 °C	(CA)8A	Poor
3	UBC-818	47 °C	(CA)8G	No band
4	UBC-826	47 °C	(AC)8C	No band
5	UBC-834	47 °C	(CA)8AG	Excellent
6	UBC-835	48 °C	(AG)8YC	Very good
7	UBC-839	49 °C	(TA)8RG	Poor
8	UBC-841	48 °C	(GA)8YC	Excellent
9	UBC-844	48 °C	(AG)8YT	Excellent
10	UBC-848	48 °C	(CA)8RG	No band
11	UBC-851	49 °C	(GT)8YG	Poor
12	UBC-852	48 °C	(AC)8T	Poor
13	UBC-854	49 °C	(TC)8RG	Excellent
14	UBC-857	49 °C	(AC)8AYG	Excellent
15	UBC-860	52 °C	(TG)8RA	Poor
16	UBC-864	48 °C	(CA)8RT	Very poor
17	UBC-865	47 °C	(CCG)6	Poor
18	UBC-873	45 °C	(GACA)4	No band
19	UBC-878	48 °C	(GGAT)4	Poor
20	UBC-879	48 °C	(CTTCA)3	Poor
21	UBC-866	55 °C	(CTC)6	Good
22	UBC-868	55 °C	(GAA)6	Excellent
23	UBC-880	48 °C	(GGAGA)3	Excellent
24	UBC-881	49 °C	(GGTG)3	No band
25	UBC-888	47 °C	BDB(CA)7	Poor

Source: Primer kit 900 (UBC 900); Single-letter abbreviations for mixed base positions: R = (A, G); Y = (C, T)

### PCR amplification and gel electrophoresis

**Fig. 2:**
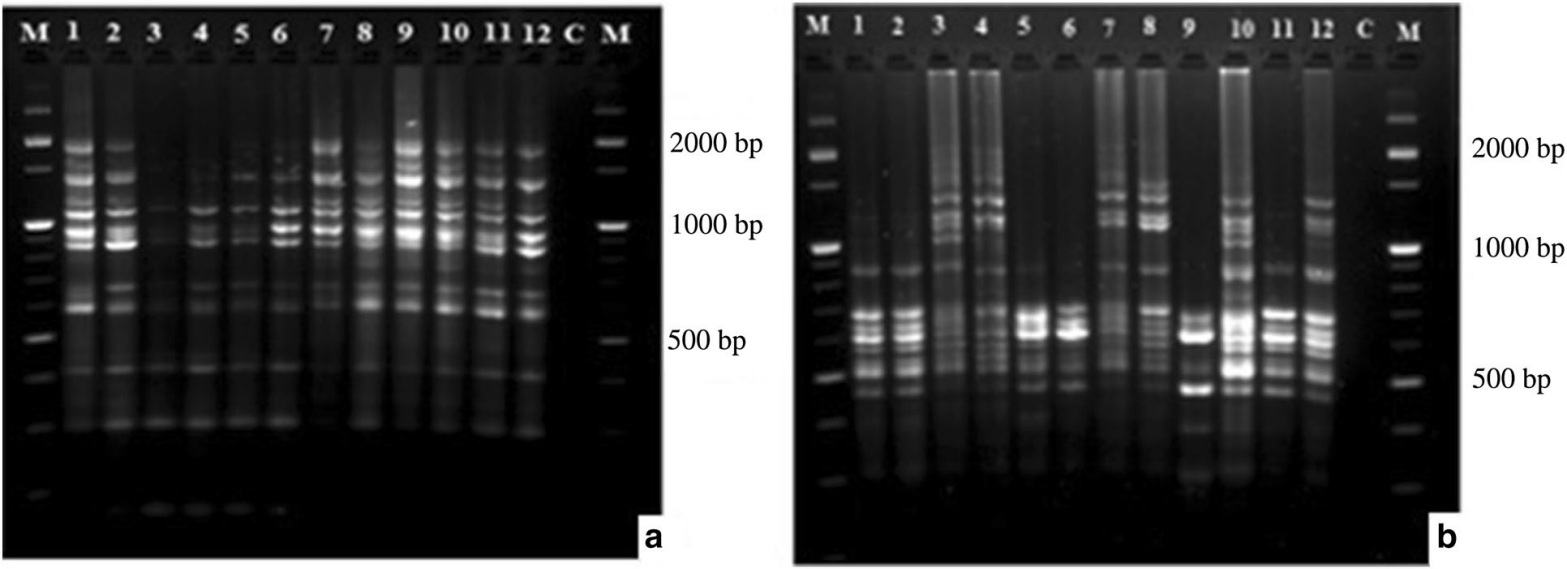
ISSR profiles generated for 12 representative individual samples of *P. edulis* populations using UBC 324 (**a**) and UBC 812 (**b**). M = 100 bp DNA ladder; C = negative control; numbers from 1 to 12 represents PE001, PE004, PE008, PE013, PE016, PE022, PE025, PE034, PE042, PE050, PE056, and PE065 accessions, respectively

PCR amplification was conducted in a final volume of 25 μl reaction mixture per sample containing ddH_2_O (15.2 μl), MgCl_2_ (25 mM) (3.0 μl), Taq buffer (10× reaction buffer S) (2.5 μl), dNTPs (1.25 mM) (1.0 μl), primer (20 pmol μl^−1^) (0.4 μl), Fire Pol DNA Polymerase (5 U μl^−1^) (0.4 μl), (FIREPOL^**®**^ DNA polymerase, Solis BioDyne, Estonia) and template DNA (10 ng µl^−1^) (2.5 µl). The amplification program was set at 94 °C for 4 min preheating and initial denaturation, followed by 40 cycles of denaturation at 94 °C for 15 s, primer annealing at specific annealing temperature for each primer (Table 2) for 1 min, extension at 72 °C for 1 min and 30 s each cycle and final extension at 72 °C for 7 min. The PCR amplification products were electrophoresed using 1.67% w/v agarose gel and size of the fragments was estimated against 100 bp DNA ladder (Thermo Fisher Scientific, Massachusetts, USA) (Fig. 2).

### Band scoring and data analysis

The resulting bands were considered as unit character and scored as present (1), absent (0) and ambiguous (?). After successful scoring, the data were assembled into a binary data matrix with the samples in a row and the ISSR markers (loci) in column.

The resulting data matrix was analyzed using appropriate software. POPGENE ver. 1.32 [19] was used in particular to determine the percentage of polymorphic loci (PPL), gene diversity (h), and Shannon’s information index (I). Arlequin ver. 3.01 [20] was used to determine the analysis of molecular variance (AMOVA) within and among the populations. Using NTSYS-pc version 2.02 [21] and Free Tree 0.9.1.50 [22], Jaccard’s similarity coefficient-based unweighted pair group method with arithmetic average (UPGMA) [23] and neighbor-joining (NJ) [24, 25] clusters were drawn to determine the genetic relationship between individual samples and populations considered in this study. To further examine the patterns of variation among individual samples, a principal coordinate analysis (PCoA) was performed using GenAlEx 6.5 [26].

## Results

### ISSR primers and their banding patterns

The ten screened and optimized ISSR primers produced 74 clear and scorable bands over the entire nine populations (an average of 7.4 per primer). The molecular weight of the fragments ranged between 200 and 2000 bp. UBC-834 produced the highest number of scorable bands (12), whereas UBC-835 and UBC-868 scored the lowest (5 bands each) (Table 3).

**Table 3:** Number of reproducible scored bands (NRSB), number of polymorphic loci (NPL), percent of polymorphic loci (PPL), Nei’s gene diversity (h), and Shannon’s information index (I) scored over the entire nine populations studied

Primers	NRSB	NPL	PPL	h ± SD	I ± SD
UBC-812	9	9	100%	0.48 ± 0.02	0.68 ± 0.02
UBC-834	12	12	100%	0.44 ± 0.03	0.63 ± 0.05
UBC-835	5	4	80%	0.39 ± 0.01	0.60 ± 0.02
UBC-841	6	6	100%	0.42 ± 0.05	0.61 ± 0.03
UBC-844	7	7	100%	0.44 ± 0.04	0.63 ± 0.04
UBC-854	7	7	100%	0.44 ± 0.04	0.63 ± 0.04
UBC-857	9	9	100%	0.41 ± 0.09	0.60 ± 0.10
UBC-866	7	5	71%	0.40 ± 0.04	0.58 ± 0.04
UBC-868	5	5	100%	0.41 ± 0.03	0.62 ± 0.03
UBC-880	7	7	100%	0.40 ± 0.10	0.59 ± 0.12
Mean	7.4	7.1	95%	0.40 ± 0.05	0.62 ± 0.05

### Evaluation of the ISSR primers and their diversity indices

The screened primers revealed that at least 91% of the loci were polymorphic throughout the entire population. Eight of the ten initially tested ISSR primers showed 100% polymorphism across the entire population, while UBC-866 showed the least percentage (71%) of polymorphic loci. The highest gene diversity (h = 0.48) and Shannon’s information index (I = 0.68) were recorded for UBC-812, followed by UBC-834, 844 and 854. UBC-835 showed the least gene diversity (h = 0.39) and UBC-866 showed the lowest Shannon’s information index (I = 0.58). Overall, there were 0.40 and 0.62, respectively, gene diversity and Shannon’s information index over the entire population (Table 3).

Among the nine populations studied, the population from Gurage administrative zone revealed the highest polymorphism (PPL = 96.08%), gene diversity (h = 0.44) and Shannon’s information index (I = 0.62), followed by population from Central Highland (PPL = 84.31%, h = 0.36 and I = 0.51). Populations from Metekel and Wolaita Sodo administrative zones showed the lowest values for PPL, h and I (Table 4).

**Table 4:** Number of individuals per population (NIPP), Number of polymorphic loci (NPL), percent polymorphism (PP), Nei’s gene diversity (h), and Shannon’s information index (I) for the nine populations recorded over the entire ten loci studied

Pop	NIPP	NPL	PPL	h ± SD	I ± SD	G_st_^a^	N_m_^a^
*Awi*	7	61	82.35	0.33 ± 0.19	0.48 ± 0.26		
*CHL*	9	62	83.78	0.36 ± 0.18	0.51 ± 0.25		
*EWo*	8	59	79.73	0.33 ± 0.19	0.48 ± 0.26		
*Gur*	8	71	95.94	0.44 ± 0.11	0.62 ± 0.15		
*GGo*	7	51	68.91	0.31 ± 0.22	0.44 ± 0.30		
*IAB*	9	52	70.27	0.30 ± 0.21	0.43 ± 0.29		
*Jim*	9	59	79.73	0.34 ± 0.19	0.49 ± 0.26		
*Met*	5	45	60.81	0.26 ± 0.22	0.38 ± 0.32		
*WSo*	5	44	59.5	0.26 ± 0.23	0.37 ± 0.32		
Mean	7.4	56	75.66	0.32 ± 0.19	0.47 ± 0.27	0.24	1.54

See Table 1 for the description

*Pop* populations

^a^N_m_ = estimate of gene flow from G_st_ where N_m_ = 0.5(1−G_st_)/G_st_

### Population genetic differentiation and distance

Hierarchical AMOVA in both without prior grouping and grouping the populations according to their collection regions revealed a significantly higher (*p* = 0.001) percentage of differentiation (92% and 96%, respectively) due to variance within populations and regions than differentiation among populations and regions (8% and 4%, respectively) (Table 5). The lower percentage of differentiation among the populations and regions was supplemented by low F_st_ (0.08 and 0.04, respectively) value (Table 5) and a higher overall gene flow (N_m_ = 1.54) (Table 4).

**Table 5:** Analysis of hierarchical molecular variance (AMOVA) without prior grouping and with grouping the populations into their collection regions

	Sources of variation	df	Sum of squares	Variance components	Percentage of variation (%)	Fixation index	*p* value
Without grouping	Among populations	8	140.6	0.93Va	8	0.08	< 0.001
	Within population	58	616.4	10.81Vb	92		
	Total	66	757.00	11.74	100		
With grouping	Among regions	3	53.73	0.48Va	4	0.04	< 0.001
	Within regions	62	703.27	11.34Vb	96		
	Total	65	757.00	11.82	100		

*df* degrees of freedom

The populations pairwise genetic distance (D) ranged from 0.11 to 0.39. In this regard, in the magnitude order, individuals from the Awi administrative zone population were relatively distantly related to the populations of Wolaita Sodo, Metekel, and Illu Aba Bora administrative zones. Similarly, population from Jimma administrative zone was relatively distant from the populations of Wolaita Sodo and Metekel administrative zones. The lowest estimate of genetic distance was observed between individuals of the Central Highland and East Wollega administrative zone populations (Table 6).

**Table 6:** Nei’s original measure of pairwise genetic distance for the nine *P. edulis* populations considered in the study (the italic values stand for the higher and lower pairwise genetic distances)

Pop	*Awi*	*CHL*	*EWo*	*Gur*	*GGo*	*IAB*	*Jim*	*Met*	*WSo*
*Awi*	****								
*CHL*	0.13	****							
*EWo*	*0.11*	*0.09*	****						
*Gur*	0.21	0.12	0.16	****					
*GGo*	0.20	0.17	0.19	0.15	****				
*IAB*	*0.34*	0.18	0.22	*0.11*	0.20	****			
*Jim*	0.15	0.18	0.19	0.15	0.18	0.29	****		
*Met*	*0.35*	0.19	0.24	0.16	0.17	0.16	*0.30*	****	
*WSo*	*0.39*	0.20	0.22	0.17	0.24	0.17	*0.32*	0.27	****

*Pop* population

**** Not applicable

### Population genetic relationships

**Fig. 3:**
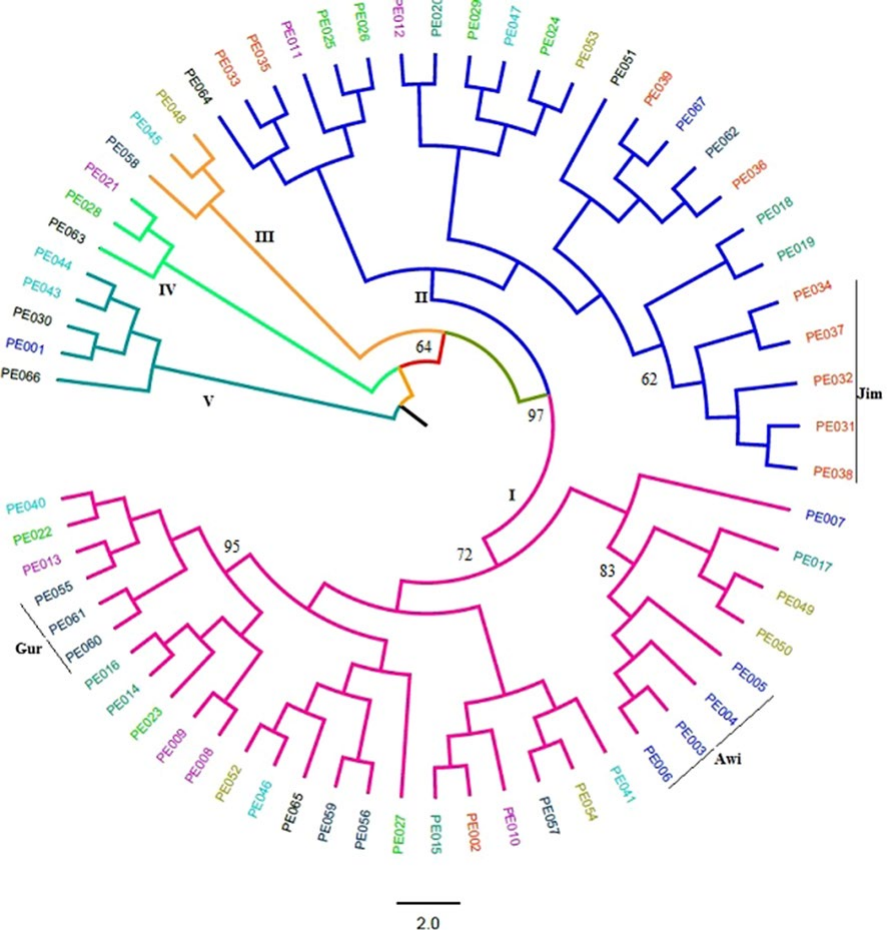
UPGMA based cluster analysis of the 66 *P. edulis* accessions where, PE represents *Plectranthus edulis* and along with numbers ranging from 001–067 stands for the individual accessions. Corresponding populations are shown in Table 1. Numbers at the roots of the branches are bootstrap values, and bootstrap values of less than 60% were not shown

**Fig. 4:**
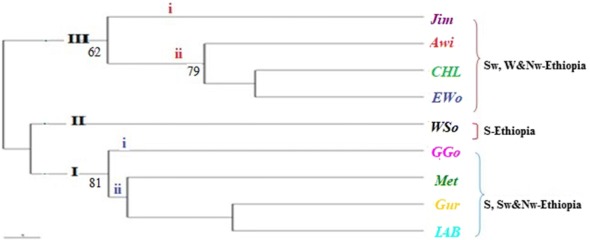
UPGMA based dendrogram for the nine *P. edulis* populations used in the study. Numbers at the roots of the branches are bootstrap values, and bootstrap values of less than 60% were not shown

Analysis of UPGMA based on Jaccard’s similarity coefficients grouped the 66 accessions into five main clusters in which several accessions (31) were grouped together under cluster **I** followed by cluster **II** (24). Clusters **III** and **IV** included three accession each, while cluster **V** contained five accessions (Fig. 3). Population level grouping, however, formed three main clusters in which populations from Jimma, Awi, Central Highland and East Wollega administrative zones were grouped together (***C3***) and populations from Gamo Gofa, Metekel, Gurage and Illu Aba Bora administrative zones were grouped together (***C1***). Population from Wolaita Sodo administrative zone appeared as a monophyletic group (***C2***) (Fig. 4). In both phylogenetic trees, we observed a weak tendency to group according to their geographical origin except some collections from Northwest Ethiopia (population from Awi administrative area) and Southwest Ethiopia (populations from Gurage and Jimma administrative areas) that formed their own mini-clusters (Fig. 3).

**Fig. 5:**
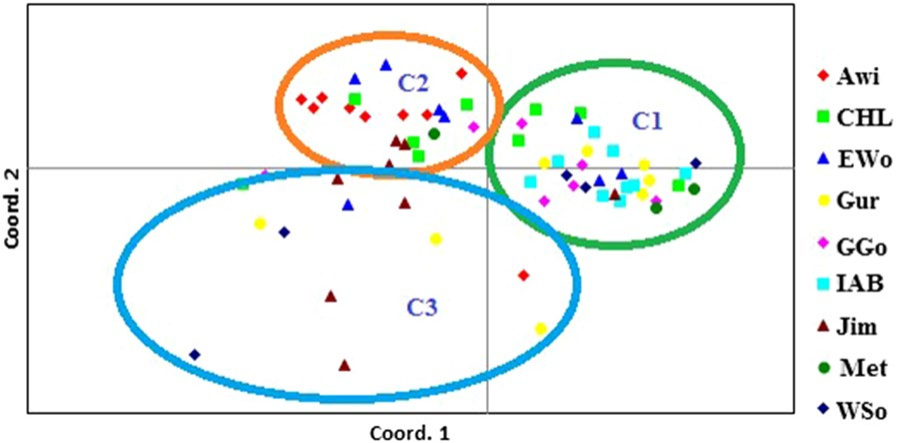
Two-dimensional PCoA representations of genetic relationships among the 67 *P. edulis* accessions based on Jaccard’s coefficients of similarity

Analysis of principal components (PCoA) based on Nei’s [27] genetic distance revealed 29.68% of the total variation for the first three (12.77%, 9.52%, 7.39%, respectively) principal axes. The 2D coordinates showed similar patterns of clustering with that of populations’ UPGMA, except for a considerable number of accessions from the administrative zones of Awi, Jimma and Illu Aba Borathat tended to form their own distinct cluster (Fig. 5).

## Discussion

### Population genetic diversity and implications for selection and conservation

ISSR marker system is one of the widely used molecular markers for assessing the extent and patterns of genetic diversity and for deducing phylogenetic relationships in a variety of crop species. Similarly, it has been used in a wide variety of plants worldwide to study genetic variation [12, 28].

The ten reproducible ISSR markers selected and used in the present study showed an overall high level of polymorphism (95%) which implies their great usefulness in revealing and evaluating the level of genetic diversity among and within *P. edulis* populations. There have been similar reports in other endemic root and tuber crops such as sweet potato [28], yam [29] and anchote [30] populations where ISSR markers showed high but varying levels of polymorphism. ISSR technique was also reported to be effective and successful in assessing genetic variability in other endemic and exotic species including lentil (*Lens culinaris medikus*) [31] and coffee (*Coffee arabica* L.) [32] from Ethiopia, Chinese grown pecan (American pecan) [33], and sesame [34].

The extent of genetic diversity in plant species is the result of one or more factors such as reproductive biology, life (evolutionary) history, geographic range of distribution, and various environmental factors that, in one way or another, affect mutation rates [35–37]. In this regard, the values in overall percent polymorphism, gene diversity and Shannon’s information index observed in *P. edulis* populations could be attributed to the crop’s clonal propagation nature in which only few parts of the population can preserve the original genetic diversity in the basal population. Furthermore, the efforts of local farmers, especially the elders, to maintain the original genetic diversity by preserving tubers over generations could be another reason for the observed higher genetic diversity indices, although the population size is rapidly declining from vast areas where it used to be widely cultivated. Twenty EST-SSR DNA markers detected a larger number of alleles and revealed a similar increasing trend in genetic diversity in twelve *P. edulis* populations that were collected from diverse agro-ecologies in the country [17].

High level of genetic variability is desirable because it increases fitness and thus reduces the likelihood of local extinction [38]. In this regard, *P. edulis* populations from Gurage, Central Highland, Awi, East Wollega and Jimma administrative zones, in order of magnitude, are important sources for improving the germplasm and taking immediate conservation actions.

On the other hand, the low values of percent polymorphism, gene diversity and Shannon’s information index observed in populations from Metekel and Wolaita Sodo administrative zones could be attributed to the smaller sample size used from these specific areas. In addition, the recent introduction and domestication of the crop to some areas, especially, the Metekel zone, which is a bit pocketed and relatively recently dominated by the Oromo and Awi ethnic groups, could be also the probable reason. Compared to other ethnic groups in the area, these ethnic groups are known to be more familiar with *P. edulis* domestication and cultivation. In agreement with this, Rampersad et al. [39] suggested that there are higher levels of maintained gene diversity in larger and older populations compared to a newly colonized habitat. This is the result of an older population having sufficient timeframe to allow mutational events to introduce new genetic variants and to decrease the effects of genetic drift thus increasing the frequency of the alleles.

### Population genetic differentiation and distance

*Plectranthus edulis* populations revealed a lower extent of genetic differentiation among the populations and the regions that could result from historical or contemporary exchange of germplasms, especially tubers for immediate planting, between or among zones and regions included in this study. The higher values of the overall gene flow (N_m_ = 1.54) and lower overall G_st_ (equivalent to F_st_) we observed could also support this premise as suggested by Wright [40] since the higher extent of gene flow (N_m_ > 1), and hence migration, is a powerful force for decreasing differentiation among populations [41].

On the other hand, the significantly higher genetic differentiation within populations and regions could be attributed to the clonal propagation nature of the crop, an important aspect in maintaining heterozygosity in the basal population over generations regardless of the current population size fluctuation. This can be explained by the fact that the crop is historically sexually reproduced through seeds, which is still rarely practiced at research sites, and this has allowed the crop to accumulate heterozygosity in the basal population to some extent. Tadele et al. [32] and Nascimento et al. [42] reported higher within population variation in clonally propagating endemic tuber crops such as anchote (*Coccinia abyssinica*) and yam, respectively. Similarly, Wodajo [43], and Seid et al. [44] reported higher levels of within population variation in safflower and *Lepidium sativum*, respectively.

The wide range of pairwise genetic distance that we detected (the highest being more than 4× of the lowest) generally indicates the high genetic variability in *P. edulis* populations that could be a valuable source for selection breeding. For this purpose, individuals from the Awi administrative zone versus Wolaita Sodo, Metekel, Illu Aba Bora and Gurage administrative zones, showing considerable pairwise genetic distance, could be used as parental sources. On the other hand, Mantel test revealed that the genetic distance did not correlate with geographic distance (data not shown) which again confirms intensive seed tuber exchange between or among farmers or the recent divergence of the crop from a common genetic base. Moulin et al. [45] reported lack of distance-related genetic variability among sweet potato landraces because of widespread practice of exchanging accessions between neighboring farmers and relatives.

### Population genetic relationship

The genetic relationships between and among *P. edulis* populations showed poor correlation between geographic origin and the patterns of clustering. Samples or populations from distant zones or regions have been found lumped together the same cluster and those from the same or nearby geographic areas have been placed under a different cluster and such grouping weakly supports the concept of “isolation by distance” [40]. Generally speaking, the clustering patterns once again denote the widely practiced tuber exchange and consequently, continuous gene flow and extensive sharing of genetic material among regions that led to reduced differentiation among the populations. However, results from other molecular marker systems such as short tandem repeats (STRs) is highly important in order to discriminate sufficiently and identify unique accessions before implementing the information for breeding and conservation.

## Conclusions

From the present study, we conclude that ISSR marker system is useful in estimating the extent of genetic diversity and generating valuable information for use in further breeding and conservation measures in indigenous crops. However, in order to generate a more reliable information, it is important to combine it with other marker systems such as agro-morphological traits-based genetic diversity assessment or co-dominant molecular marker systems, such as SNPs, which have a higher potential for estimating population genetic structure.

On the basis of genetic diversity indices such as gene diversity, Shannon’s information index, and percent of polymorphic loci, Gurage, Central Highland, Awi, East Wollega and Jimma administrative zones are relatively better areas for *P. edulis* diversification and conservation. The higher gene flow among the different regions, coupled with the current decline in population size, may result in loss of valuable agronomic traits unless counteracting action is taken.

Representative samples from all potential growing areas need to be collected exhaustively to provide a good estimate of the crop’s existing genetic diversity for use in improving it and reversing its current rapid genetic erosion.

## Data Availability

Not applicable.

## Abbreviations

AMOVA: analysis of molecular variance
CTAB: cetyl trimethyl ammonium bromide
ISSR: inter-simple sequence repeats
SNNPs: Southern Nations, Nationalities, and Peoples’ Region
PCoA: principal coordinates analysis
UPGMA: unweighted pair group with arithmetic mean
